# Editorial: Applied Genetics of Natural Fiber Plants

**DOI:** 10.3389/fgene.2021.647225

**Published:** 2021-04-13

**Authors:** Sylvain Niyitanga, Xin Yang, Gea Guerriero, Shuangxia Jin, Jianmin Qi, Liemei Zhang, Liwu Zhang

**Affiliations:** ^1^Key Laboratory of Ministry of Education for Genetics, Breeding and Multiple Utilization of Crops/Fujian Key Laboratory for Crop Breeding by Design, College of Agriculture, Fujian Agriculture and Forestry University, Fuzhou, China; ^2^Experiment Station of Ministry of Agriculture and Rural Affairs for Jute and Kenaf in Southeast China/Public Platform of Fujian for Germplasm Resources of Bast Fiber Crops, Fujian Agriculture and Forestry University, Fuzhou, China; ^3^Environmental Research and Innovation Department, Luxembourg Institute of Science and Technology, Hautcharage, Luxembourg; ^4^National Key Laboratory of Crop Genetic Improvement, Huazhong Agricultural University, Wuhan, China

**Keywords:** fiber plants, genetics, genomics, breeding, next-generation sequencing technology

## Introduction

Fiber crops are plants grown for their cellulosic fibers, which are used for textile products and other many industrial purposes, such as substitutes of man-made fibers in biocomposites (Andre et al., 2016). Natural fiber plants include cotton, flax, jute, kenaf, hemp, ramie, sisal, nettle, and others. These crops have received much consideration from breeders owing to their agronomic features (namely fast growth), sustainability, economic significance, and others. Desired agronomic features of fiber plants, notably fiber quality, abiotic, and biotic stress resistance are major targets for modern breeding. Consequently, modern methods for molecular breeding have been used not just to improve these traits, but also to meet the global consumers' demand and bring convenience to growers. Next-Generation Sequencing (NGS) tools have extensively hastened the improvement of genome and transcriptome sequencing, as well as re-sequencing of fiber crops. The assembled genome of fiber plants such as flax Dmitriev et al., kenaf (Zhang et al., 2020) and others can aid scientists to detect the precise position of genes of interest on chromosomes. The identification of these associated markers constitutes the foundation of the genotyping technology and is useful to determine the genetic relatedness and structure analysis among different accessions (Zhang et al., 2020). These, in their turn are the foundation of genetic mapping and MAS (molecular marker-assisted selection). The objective of this editorial is to condense recently available studies on the genome sequencing of fiber-producing plants and genome-based techniques for gene identification and molecular breeding, which can provide a valuable resource for modern fiber crops' genetic improvement.

## The Genome Sequencing of Fiber Crops

The work of genome sequencing in plants has greatly advanced the existing genomic knowledge of the past years. The complete genome sequencing data can be utilized to analyze the whole genome of species and elucidate gene functions. It works as an efficient approach for disclosing the mechanism of plant development, growth and differentiation at the molecular level. The whole genome sequence of fiber-producing plants should offer a better insight into fiber biogenesis and fiber-linked molecular processes and could help in producing genetically engineered plant lines that possess superior agricultural features. The advancement of NGS high-throughput technologies has made plant genome sequencing studies affordable and relatively easy. Therefore, several studies have focused on the genome and transcriptome sequencing of various fiber crops. Very recently, a high-quality genome sequence (361.7 Mb) of flax (*Linum usitatissimum* L.) has been assembled by Oxford Nanopore and Illumina throughputs Dmitriev et al. Other sequenced fiber plants include jute (Islam et al., 2017), hemp (Van Bakel et al., 2011), cotton (Li et al., 2015), ramie (Liu et al., 2018), nettle (Xu et al., 2019). The use of NGS techniques will also be useful in metagenomics studies addressing retting, e.g., in flax Djemiel et al..

## Gwas in Fiber Crops

Genome-wide association study (GWAS) is a useful technique centered on the association between phenotypes and genotypes in a group of accessions, to investigate the basis of quantitative trait variation by considering the interaction between the environment and genetic factors (Saïdou et al., 2014). GWAS can be performed by gathering the various wild and cultivated accessions to sequence and compare the genomic discrepancy among these accessions, particularly traits influenced by many factors. The knowledge of the genetic structure of a plant's fiber quality is one of the primary requisites for breeding new cultivars with enhanced fiber quality. Recently, in a GWAS comprising 123 *Cannabis sativa* accessions, molecular markers containing the major QTLs and candidate genes for regulating diverse fiber quality traits, such as the content of lignin, glucose and others have been identified Petit et al. These will be useful for molecular breeding for high fiber quality in textile hemp. However, markers identified with GWAS may produce incorrect positives and very low reproducible markers in other accessions. Consequently, markers produced by GWAS necessitate further polymorphism amplification or genotyping for further confirmation in other natural fiber crops.

## Genome-Based Molecular Markers and Gene Identifications for Fiber Crop Breeding

Owing to the high request for fiber crops ensuring high yield and quality, marker-assisted selection (MAS) has become a key breeding approach centered on genotype analysis to enhance productivity. Polymorphisms and sufficient molecular markers are some of the major factors for MAS breeding. Formerly, when complete genome sequences were not available, molecular markers such as RLFP were identified; nevertheless, these markers have relatively low polymorphism and inadequate numbers. With the availability of reference genomes and advanced NGS tools, the number of molecular markers such as SSRs, InDels, SNPs has been improved significantly in natural fiber. Very recently, genome sequences of *Cannabis* species were used to develop SSR markers (Zhang et al., 2020) and transcriptomics data on specific flax bast fibers' developmental stages helped in the identification of candidates that could be used to improve fibers' quality Galinousky et al. Also, genome sequencing data can be used to identify genes linked to important traits Yang et al., which is the key to MAS breeding.

## Gene Transformation for Fiber Crop Breeding.

Gene transformation is another important approach for creating genetic diversity in plant species, which is one of the major factors for MAS breeding. Using this approach, a candidate gene with superior traits is selected from a given donor plant species, cloned and then integrated into the genome of another plant species for its expression. This technique enables scientists to genetically engineer plants with superior traits. In order to improve fibers' traits, it is essential to identify promoters driving the expression of the gene in the target tissue. For example, genes highly expressed in ramie bark have been isolated and their promoters sequenced. Constructs in which the reporter gene *GUS* was placed under the control of the identified promoters were introduced in *Arabidopsis* to confirm the expression in the phloem Wang et al. These data can serve to devise genetic engineering techniques aimed at improving the bast fibers' properties of fiber crops.

## Author Contributions

All authors listed have made a substantial, direct and intellectual contribution to the work, and approved it for publication.

## Conflict of Interest

The authors declare that the research was conducted in the absence of any commercial or financial relationships that could be construed as a potential conflict of interest.
